# The U-Shape Relationship between Triglyceride-Glucose Index and the Risk of Diabetic Retinopathy among the US Population

**DOI:** 10.3390/jpm13030495

**Published:** 2023-03-09

**Authors:** Yu Zhou, Qiong Lu, Min Zhang, Ling Yang, Xi Shen

**Affiliations:** 1 Department of Ophthalmology, Ruijin Hospital, LuWan Branch, Shanghai Jiao Tong University School of Medicine, Shanghai 200020, China; 2 Department of Ophthalmology, Ruijin Hospital, Shanghai Jiao Tong University School of Medicine, Shanghai 200020, China

**Keywords:** diabetes mellitus, diabetes retinopathy, triglyceride–glucose index, National Health and Nutrition Examination Survey

## Abstract

Objective: To explore the association of diabetic retinopathy (DR) with TyG index and TyG-related parameters among the United States population. Methods: This cross-sectional study is conducted in adults with diabetes mellitus based on the National Health and Nutrition Examination Survey (NHANES) from 2005 to 2018. Multivariate logistic regression, restricted cubic spline, trend test, receiver operating characteristic curve and subgroup analysis are adopted to uncover the association of DR with TyG index and TyG-related parameter levels in diabetics. Results: An aggregate of 888 eligible participants with diabetes is included, involving 263 (29.6%) patients with DR. The participants are stratified according to the quartile of TyG index and TyG-related parameters (Q1–Q4). Following the adjustments of the confounding factors, a multivariate logistic regression analysis finds that TyG-BMI, TyG index and Q4-TyG index are significant risk factors for DR. The restricted cubic spline shows that TyG index and the DR risk of diabetes patients are proved to be U-shaped related (*p* for nonlinearity = 0.001). Conclusions: The triglyceride-glucose index has a U-shaped correlation with the risk of diabetic retinopathy, which has potential predictive value.

## 1. Introduction

The prevalence of diabetes is increasing worldwide due to rapid population aging and unhealthy lifestyles characterized by smoking, excessive drinking, sedentary behavior and high-calorie diet intake. Diabetes retinopathy (DR) is a pervasive microvascular complication of diabetes that often leads to blindness, with a global prevalence of 34.6% [1]. The pathological changes of DR are often concealed, leading to delayed medical intervention, and advanced stages of the disease, which results in irreversibly impaired vision and unfavorable treatment prognosis. Consequently, early prediction, diagnosis and treatment of DR hold significant clinical importance.

Currently, the pathogenesis of DR remains insufficiently understood, and hyperglycemia is typically regarded as the primary cause of DR. In the past, the prevention and treatment of DR focused mainly on managing blood glucose levels and glycosylated hemoglobin levels in diabetic patients. Nevertheless, the pathogenesis of DR is a multifaceted process, and several mechanisms and factors contribute to its occurrence and progression, such as hypertension, abnormal lipid metabolism, inflammation and insulin resistance [2,3,4].

At present, the hyperinsulinemic normoglycemic clamp (HIEC) is considered the gold standard for assessing insulin resistance [5], as it measures peripheral tissue sensitivity to insulin. Nonetheless, due to its complexity and cost, this technique is not widely used in clinical practice. Alternatively, the homeostasis model assessment of insulin resistance (HOMA-IR) is the most commonly used method for assessing insulin resistance in clinical practice and has a good correlation with HIEC [6]. However, fasting insulin needs to be measured when calculating HOMA-IR, which can be difficult to obtain in some primary medical institutions. In 2008, it was first reported that the triglyceride–glucose (TyG) index could be used as a substitute index for insulin resistance [7], which does not rely on fasting insulin levels. Compared to other insulin resistance evaluation indexes, the advantages of the TyG index lie in its lower cost, simpler operation and wider applicability.

In recent years, numerous studies have corroborated the relationship between the TyG index and the risk of IR-related metabolic diseases such as diabetes [8], nonalcoholic fatty liver disease (NAFLD) [9], cardiovascular disease [10] and metabolic syndrome [11]. What is more, obesity is commonly acknowledged to trigger or worsen the presence of insulin resistance [12]. Several studies have evaluated that TyG-related parameters are more effective than the isolated TyG index [13], such as TyG combined with body mass index (TyG-BMI), TyG combined with waist circumference (TyG-WC) and TyG combined with waist-height ratio (TyG-WHtR). However, the effect of applying the TyG index and TyG-related parameters to predict the risk of diabetic retinopathy in diabetes patients is still unclear. Therefore, this study aims to determine the predictive value of the TyG index and TyG-related parameters for the DR among the US population with diabetes.

## 2. Materials and Methods

### 2.1. Data Source

**Figure 1:**
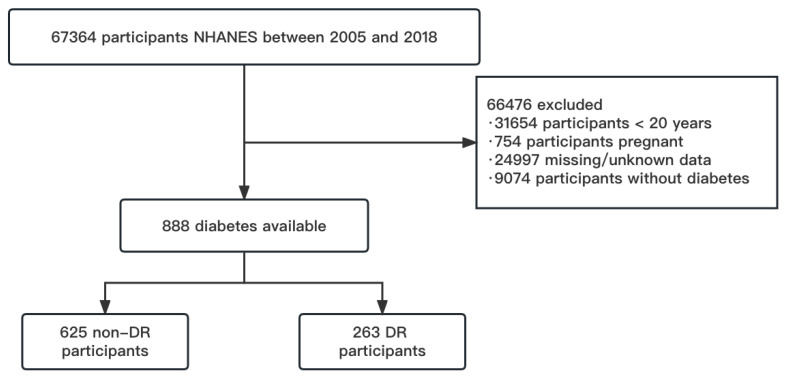
The participant screening flow diagram.

The National Health and Nutrition Examination Survey (NHANES) is a nationwide cross-sectional study aimed at assessing the health and nutrition status of the general population of the United States, using a complex sampling strategy. National Center for Health Statistics granted the study procedures of the Ethics Review Board (Protocol #2005-06, #2011-17, #2018-01). Informed consent of the participants was obtained before collecting any data. Centers for Disease Control and Prevention (CDC) provided health statistics and details of the NHANES protocol [14]. All participants were required to take part in standardized home interviews which obtained demographic and health related issues, meanwhile comprehensive physical and laboratory examinations were carried out at the mobile examination center (MEC). In our study, we used seven cycles of the open NHANES database (2005–2018). Figure 1 depicts the selection process. Missing data and subjects younger than 20 years of age or pregnant or without diabetes, are excluded. For more information on the data, please visit www.cdc.gov/nchs/nhanes/ (accessed on 29 October 2022).

### 2.2. Data Source

During the home interview, in the face of the question “have you ever been told by a doctor or health professional that you have diabetes or sugar diabetes?”, the participants who answer “yes” were defined as diabetes patients. Digital images of the retina, obtained using Topcon non-mydriatic fundus photography (TRC-NW6S, Topcon, Tokyo, Japan) in the 2005–2006 and 2007–2008 cycles, were sent to contract graders of the University of Wisconsin-Madison for reading. DR participants included those diagnosed by retina image and self-reported DR individuals.

### 2.3. Study Variables

Information on age, gender, race/ethnicity, education, history of comorbidities (coronary heart disease (CHD), stroke, hypertension and nephropathy) and ratio of family income to poverty (PIR) was collected through demographic questionnaires in family interviews. The height, waist circumference (WC) and weight of all participants were collected by trained health technicians at the mobile examination center (MEC). Body mass index (BMI) was calculated by the following formula: BMI = body weight (kg)/height (m2) [15]. Participants’ had fasting venous blood drawn after at least an 8-h overnight fast, and the measurements, including high-density lipoprotein (HDL, mg/dL), low-density lipoprotein (LDL, mg/dL), total cholesterol (TC, mg/dL), triglyceride (mg/dL), fasting glucose (mg/dL), fasting insulin (uU/mL) and glycohemoglobin (HbA1c, %) were obtained.

TyG index is equal to ln [triglyceride (mg/dL) × glucose (mg/dL) ÷ 2] under fasting conditions [16],

HOMA-IR = fasting glucose (mmol/L) × fasting insulin (uU/mL)/22.5 [6],

TyG-WC = TyG index × waist circumference,

TyG-BMI = TyG index × body mass index,

waist-to-height ratio (WHtR) = WC/height,

TyG-WHtR = TyG index × WHtR [17].

### 2.4. Statistical Analysis

A χ2 test and independent sample *t*-test were used to compare the differences in the characteristics between categorical variables and continuous variables at baseline in the non-DR group and DR group, respectively. Continuous variables are represented as mean ±standard deviation, and the categorical variables are shown as frequencies. A logistic regression analysis was carried out to evaluate the relationship between the risk of DR and TyG-related parameters, and to calculate the odds ratio (OR) and 95% confidence interval (CI), which showed the outcomes of several models modifying confounding factors. Among them, the crude model did not include any adjustment for covariates, model 1 adjusted the general demographic variables, and model 2 added HDL, LDL, TC, hypertension history and retinopathy history on the basis of model 1. In addition, the tendency test was conducted with the first quartile as a reference. Restricted cubic splines (RCSs) were used to identify nonlinear relationships. The diagnostic efficacy of the TyG index and its related parameters for DR were analyzed and drawn using the receiver operating characteristic (ROC) curve, evaluating the screening value of each method by the area under the ROC curve (AUC). A hierarchical logistic regression model carried out an exploratory hierarchical analysis on some subgroups and determined whether interactions occurred. *p* < 0.05 (bilateral) was considered to have statistical significance. All analyses were conducted through R language 4.2.2 and SPSS 22.0.

## 3. Results

### 3.1. Baseline Characteristics of the Participants

The study samples include 888 adults, 443 females and 445 males. The average age is 62.2 ± 12.1 years. Two-hundred and sixty-three (29.6%) patients have DR. Among the diabetes patients, only 13.7% have a normal BMI, and 85.6% are overweight or obese. Table 1 shows the comparison between non–DR and DR adults. The result shows that the glucose, HbA1c and TyG index of the DR participants increase. Meanwhile, it suggests participants with retinopathy, CHD and stroke history are more likely to have DR. Age, race, education, PIR, LDL, HDL, triglyceride, total cholesterol, insulin, waist circumference, body mass index and hypertension history show no significant differences between diabetic patients with/without DR.

### 3.2. Logistic Regression Analyses for the Relationship between Various TyG-Related Parameters and DR in Different Models

The logistic regression model depicts the relationship between the various TyG-related parameters and DR, as shown in Table 2. In the crude model, the TyG index (OR 1.412, 95% CI 1.024–1.947, *p* = 0.035) and Q4-TyG index (OR 1.603, 95% CI 1.069–2.404, *p* = 0.022) are important risk factors for DR. The ORs of DR increase along with the TyG index quartiles (*p* for trend = 0.027). Following an adjustment for the demographic characteristics (age, sex, race, education, PIR), model 1 shows that the ORs are 1.603 (95%CI 1.146–2.242, *p* = 0.006) and 1.826 (95% CI 1.200–2.779, *p* = 0.005) for the TyG index and Q4-TyG index, respectively. Based on our exploration, there are dose-response relationships between the quartiles of the TyG index which takes the first quartile as a reference and the risk of DR (*p* for trend = 0.004). This trend remains significant even after further modification of the confounding factors (HDL, LDL, TC, hypertension and retinopathy) in model 2 (*p* for trend = 0.002). Upon modifying potential confounding variables (model 2), the ORs of DR are 1.182 (95% CI 0.756–1.848), 1.327 (95% CI 0.831–2.121) and 2.186 (95% CI 1.323–3.613) for the second, third and fourth TyG index quartile, respectively. TyG-BMI becomes an unignorable risk factor for DR (OR 1.014, 95% CI 1.001–1.027, *p* = 0.035), TyG index (*p* = 0.002) and Q4-TyG index (*p* = 0.002) remain critical risk factors for DR.

### 3.3. Restricted Cubic Splines for the Relationship between the TyG Index and DR

**Figure 2:**
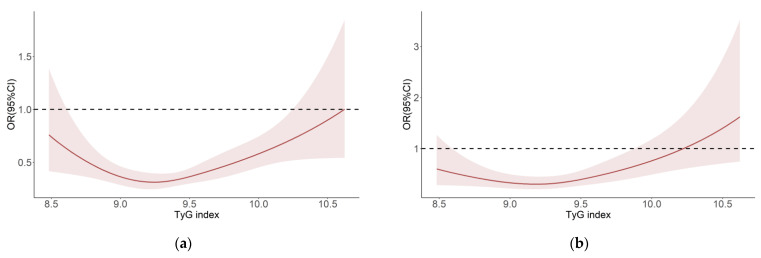
The association between the baseline TyG level and DR based on restricted cubic splines. The odd ratios for DR (solid line) and 95% confidence intervals (shaded portion) are presented. (**a**) Correlation between DR and TyG index in the crude model. (**b**) Correlation between DR and TyG index adjusts for age, gender, race, education, PIR, HDL, LDL, TC, hypertension history and retinopathy history.

An approximately U-shaped association between the TyG index and DR, demonstrated and modeled by the restricted cubic splines with four knots, is displayed among diabetes participants, which suggests that the TyG index is non-linearly associated with DR participants (Figure 2). In the crude model (Figure 2a), when the TyG index is greater than 9.21, the risk of DR increases (*p* for non-linearity = 0.001). Following further adjustments of confounding factors (Figure 2b), this diagram demonstrates a reduction of the risk of DR when the TyG index is beneath 9.18, then it increases afterward (*p* for non-linearity = 0.001).

### 3.4. Subgroup Analysis of the Correlation between the TyG Index and DR

To verify the impact of the TyG index on DR, the study examined the interaction terms of effective variables that may lead to changes in DR risk. A subgroup analysis was conducted according to demographic factors, laboratory examination, history of hypertension (yes or no) and history of kidney disease (yes or no). Table 3 shows the results of a subgroup analysis of the correlation between the TyG index and the risk of DR, there is no difference in the TyG index among most pre-specified subgroups in DR participants, except for gender (*p* for interaction = 0.013), total cholesterol (*p* for interaction = 0.013) and retinopathy history (*p* for interaction = 0.032). The TyG index has a significant interaction relationship with DR in the female group (OR 2.669, 95% CI 1.395–5.109), high total cholesterol group (OR 1.004, 95% CI 1.001–1.006) and retinopathy history group (OR 2.096, 95% CI 1.328–3.307) after adjusting for the confounding variables.

In addition, according to the history of coronary heart disease (CHD) and stroke, diabetic patients are classified into vasculopathy(n = 171) and non-vasculopathy groups (n = 717). Table 4 shows the results of the vasculopathy subgroup analysis of the correlation between the TyG-related parameters and the risk of DR after adjusting for the confounding factors in model 2. The TyG index is a risk factor for a DR event in participants without vasculopathy (OR:2.656, 95% CI: 1.643–4.294, *p* < 0.01).

### 3.5. Diagnostic Efficacy of Various Parameters for DR

**Figure 3:**
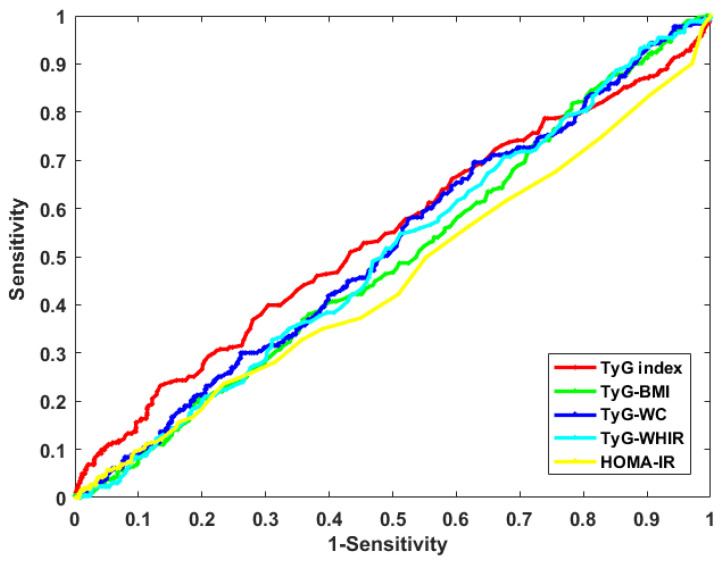
The ROC curves of the TyG index, TyG-WC, TyG-BMI, TyG-WHtR and HOMA-IR for diagnosing DR. Abbreviation: TyG: triglyceride–glucose, TyG-BMI: TyG combined with body mass index, TyG-WC: TyG combined with waist circumference, TyG-WHtR: TyG combined with waist-height ratio, HOMA-IR: homeostasis model assessment of insulin resistance.

Using a receiver operating characteristic (ROC) curve to analyze the diagnostic efficacy of the TyG index, TyG-WC, TyG-BMI, TyG-WHtR and HOMA-IR for DR (Figure 3). The optimum cut-off value of the TyG index for DR diagnosing is 9.86 (AUC 0.543, sensitivity = 23.2%, specificity = 86.56%). In addition, the study also calculates the best cut-off value of TyG-WC as 961.0 (AUC = 0.517, sensitivity = 69.6%, specificity = 37.3%). Moreover, the sensitivity, specificity, AUC and the best cut-off value of TyG-BMI to diagnose DR are 81.8%, 21.8%, 0.494 and 247.3, respectively. The sensitivity, specificity, AUC and the best cut-off value of TyG-WHtR are 93.16%, 11.04%, 0.504 and 5.05. While the sensitivity, specificity, AUC and the best cut-off value of HOMA-IR are 5.7%, 94.9%, 0.454 and 41.04, respectively. An AUC greater than 0.5 is considered to have diagnostic applications, these results show that the diagnostic value of the TyG Index for DR patients is higher than that of TyG-WC, TyG-BMI, TyG-WHtR and HOMA-IR.

## 4. Discussion

This research further explores the relation and application of the TyG index and its related parameter in DR patients, using the NHANES database based on the national representative population distributed throughout the United States. This study finds that the TyG-BMI, TyG index and Q4-TyG index are significant risk factors for DR. Additionally, the TyG index exhibits a significant dose-response relationship with DR risk. Notably, this study is the first to demonstrate a U-shaped relationship between the TyG index and DR risk after adjusting for confounding factors, and the risk of DR bottoms out when the TyG index is approximately 9.18. These findings suggest that the TyG index is a robust indicator of DR risk and can facilitate the identification and monitoring of diabetic patients who are at risk for DR.

Research has explored the relation between the TyG index and DR. A nested case-control study carried out by Yao et al. on Chinese T2DM inpatients shows that the TyG index is highly correlated with severe levels of DR [18]. However, another study found that the rise of the TyG index was intimately related to microalbuminuria and the risk of cerebrovascular disease, which is irrelative to DR [19]. Diverse research designs, sample sizes and statistical methods may account for the discrepancies in research findings. Notably, the aforementioned studies primarily focus on inpatients, thus necessitating further validation from the community population.

The TyG index has been reported to be a composite biochemical indicator that reflects the combined effect of glucose and lipids [20]. The relationship between DR and abnormal glycolipid metabolism indicators has been extensively discussed [21,22]. DR is a progressive eye disease that poses a threat to vision. Hyperglycemia damages the retinal microvascular system, leading to diabetic macular edema (DME), neovascularization, tractive retinal detachment, vitreous hemorrhage and ultimately blindness [23]. In spite of hyperglycemia as the core of diabetic retinopathy development, abnormal lipid metabolism exacerbates the condition [24]. Montgomery et al. observed that a severe decrease in b/a wave ratio and retinal function is considered to be caused by dyslipidemia and its related lipid oxidation and increased oxidative stress [25]. Therefore, lipid abnormalities and oxidative stress likely aggravate the damage caused by diabetes to the retina. However, experiments conducted by Acharya et al. on rats revealed that hyperglycemia and aging exacerbate inflammation and oxidative stress induced by dyslipidemia [26]. The research indicates an intertwined pathogenesis between abnormal glucose and lipid metabolism.

The TyG index is used as a substitute measurement for evaluating insulin resistance [27]. Insulin resistance is mainly manifested by decreased insulin sensitivity, which is prevalent in a variety of metabolic-related diseases. Insulin resistance runs throughout diabetes, and many studies have demonstrated that chronic low-level inflammation due to obesity promotes the occurrence and development of diabetic complications by aggravating insulin resistance [28]. Obesity is one of the established risk factors for diabetes mellitus [29], and it is characterized by abnormal or excessive fat accumulation. In this study, 85.6% of diabetic patients are overweight or obese (BMI ≥25 kg/m^2^) [30]. Previous studies show that hypertrophy or an increased number of adipose cells results in the enhanced or weakened expression of its secreted hormones and adipokines, which affects the effects of insulin from different levels, and further induces or exacerbates the presence of insulin resistance [31,32]. These possibly explain the mechanism of the TyG index related to diabetes retinopathy, but the specific mechanism still needs further study.

Multiple studies have demonstrated that combining the TyG index with obesity-related indicators, such as waist circumference, BMI, and WHtR, enhances the ability to predict insulin resistance. A large-scale cross-sectional study concluded that TyG-BMI shows the best discriminative power for assessing insulin resistance in clinical settings [33]. Taiwo et al. concluded that TyG-WHtR is a superior predictor of metabolic syndrome risk in Nigerians compared to the TyG index and other TyG-related parameters [34]. In contrast, another study indicated that the TyG index is the better predictor of coronary heart disease risk and coronary atherosclerosis severity in NAFLD patients compared to the TyG-BMI [17]. This study found that the TyG-BMI and TyG index are important risk factors for DR after correcting for related confounders.

Our study showcases the initial evidence of a U-shaped nonlinear relationship between the TyG index and DR. Furthermore, even after controlling for confounding factors, a significant correlation between the TyG index and DR persists. This discovery will be of great help to clinicians, as it suggests that the TyG index could potentially serve as a straightforward, reliable and practical measure in the treatment and management of DR. Our subgroup analysis also indicates that IR-related diabetic retinopathy may affect female diabetic patients more acutely. However, this does not necessarily imply a higher prevalence of female patients. Therefore, clinicians should pay more attention to insulin resistance in female diabetic patients during clinical practice, while also taking into account the blood lipids and kidney status. Moreover, the vasculopathy subgroup analysis highlights the TyG index as a critical risk factor in diabetic patients without vasculopathy. As the TyG index could detect retinal damage earlier than cardiovascular and cerebrovascular damage in diabetic patients, monitoring this index during the disease could help reduce the risk of incident DR and resultant healthcare burdens. Nonetheless, the complexity of the disease and the presence of numerous combined risk factors in diabetic patients with vasculopathy may weaken the correlation of the TyG index. Future studies should thus aim to determine the safety threshold of the TyG index to guide medication and treatment in diabetic individuals, thereby delaying the onset and progression of diabetic retinopathy.

This study also has some limitations. (1) This is a cross-sectional study. Therefore, it can only illustrate a correlation between DR and the TyG index, but it requires further prospective research to clarify the causal relationship. (2) The lack of DR severity and other residual confounding factors that are difficult to measure or evaluate probably affect our conclusions [35]. However, these limitations might be balanced by our strengths, including the large sample size, diverse ethnicities in the United States, wide age range, precise data and information on covariates, etc.

## Figures and Tables

**Table 1:** Baseline characteristics of diabetes in the NHANES (2005–2018).

Characteristics	Non-DR (n = 625)	DR (n = 263)	*p* Value
Age, mean	61.72 ± 12.29	63.21 ± 11.75	0.095
Gender, n (%)			0.037 *
Male	299 (47.8%)	146 (55.5%)	
Female	326 (52.2%)	117 (44.5%)	
Race, n (%)			0.124
White	253 (40.5%)	104 (39.5%)	
Black	161 (25.8%)	84 (31.9%)	
Other	211 (33.8%)	75 (28.5%)	
Education, n (%)			0.494
<high school	217 (34.7%)	103 (39.2%)	
≥high school	408 (65.3%)	160 (60.8%)	
PIR	2.29 ± 1.54	2.23 ± 1.46	0.600
Glucose (mg/dL)	149.89 ± 56.32	166.09 ± 73.62	0.000 **
HbA1c, %	7.18 ± 1.66	7.89 ± 1.90	0.000 **
LDL (mg/dL)	100.65 ± 36.22	99.79 ± 35.37	0.744
HDL (mg/dL)	50.04 ± 13.35	50.35 ± 13.69	0.912
Triglyceride (mg/dL)	140.54 ± 69.38	141.57 ± 68.23	0.839
TC (mg/dL)	178.82 ± 41.70	178.24 ± 41.26	0.850
Insulin (uU/mL)	18.78 ± 31.86	15.98 ± 20.92	0.190
TyG index	9.42 ± 0.41	9.49 ± 0.52	0.035 *
WC (cm)	108.64 ± 15.65	108.73 ± 15.69	0.941
BMI, kg/m^2^	32.07 ± 7.07	31.79 ± 6.80	0.466
Hypertension, n (%)			0.516
Yes	422 (67.5%)	186 (70.7%)	
No	203 (32.5%)	77 (29.3%)	
Renopathy, n (%)			0.004 **
Yes	49 (7.8%)	37 (14.1%)	
No	576 (92.2%)	226 (85.9%)	
CHD, n (%)			0.005 **
Yes	57 (9.1%)	41 (15.6%)	
No	568 (90.9%)	222 (84.4%)	
Stroke, n (%)			0.042 *
Yes	57 (9.1%)	36 (13.7%)	
No	568 (90.9%)	227 (86.3%)	

Abbreviations: PIR poverty income ratio, HDL high density lipoprotein, LDL low density lipoprotein, TC total cholesterol, TyG index triglyceride–glucose index, BMI body mass index, WC waist circumference, CHD coronary heart disease, * *p* < 0.05, ** *p* < 0.01.

**Table 2:** Logistic regression analysis for the relationship between various TyG-related parameters and DR in different models.

Parameters	Crude Model OR (95%CI), *p* Value	Model 1 OR (95%CI), *p* Value	Model 2 OR (95%CI), *p* Value
TyG index	1.412 (1.024–1.947), 0.035 *	1.603 (1.146–2.242), 0.006 **	1.946 (1.282–2.954), 0.002 **
TyG index quartile			
Q1	reference	reference	reference
Q2	1.089 (0.716–1.656), 0.690	1.100 (0.718–1.683), 0.662	1.182 (0.756–1.848), 0.463
Q3	1.089 (0.716–1.65 6), 0.690	1.162 (0.759–1.777), 0.490	1.327 (0.831–2.121), 0.236
Q4	1.603 (1.069–2.404), 0.022 *	1.826 (1.200–2.779), 0.005 **	2.186 (1.323–3.613), 0.002 **
*p* for trend	0.027 *	0.004 **	0.002 **
TyG–BMI	1.000 (0.997–1.002), 0.662	1.000 (0.998–1.003), 0.821	1.014 (1.001–1.027), 0.035 *
TyG-BMI quartile			
Q1	reference	reference	reference
Q2	1.111 (0.743–1.663), 0.608	1.141 (0.758–1.717), 0.529	1.118 (0.728–1.910), 0.502
Q3	0.957 (0.636–1.441), 0.835	1.052 (0.690–1.604), 0.813	1.135 (0.613–2.104), 0.686
Q4	0.916 (0.607–1.381), 0.916	1.033 (0.667–1.599), 0.884	1.151 (0.444–2.989), 0.772
*p* for trend	0.532	0.973	0.759
TyG-WC	1.000 (0.999–1.001), 0.563	1.000 (0.999–1.001), 0.479	1.000 (0.999–1.001), 0.510
TyG-WC quartile			
Q1	reference	reference	reference
Q2	0.978 (0.649–1.474), 0.917	1.049 (0.691–1.592), 0.822	1.032 (0.674–1.580), 0.884
Q3	1.000 (0.664–1.505), 1.000	1.030 (0.677–1.567), 0.890	1.008 (0.654–1.554), 0.972
Q4	1.089 (0.726–1.634), 0.679	1.138 (0.745–1.737), 0.549	1.106 (0.708–1.728), 0.657
*p* for trend	0.669	0.591	0.701
TyG-WHtR	0.989 (0.845–1.157), 0.887	1.052 (0.891–1.241), 0.552	1.048 (0.879–1.249), 0.599
TyG-WHtR quartile			
Q1	reference	reference	reference
Q2	0.960 (0.636–1.449), 0.845	1.018 (0.667–1.553), 0.935	1.037 (0.672–1.598), 0.871
Q3	1.219 (0.815–1.822), 0.335	1.339 (0.884–2.028), 0.168	1.388 (0.903–2.134), 0.135
Q4	0.935 (0.618–1.415), 0.751	1.078 (0.697–1.666), 0.735	1.070 (0.672–1.704), 0.776
*p* for trend	0.937	0.449	0.466

Abbreviations: TyG index triglyceride–glucose index, BMI body mass index, WC waist circumference, WHtR waist-to-height ratio. Data are presented as odds ratios, 95% confidence intervals and *p*-value. The quartile cut-off values of the baseline TyG index are ≤9.14, 9.14~9.41, 9.41~9.69 and ≥9.69. The quartile cut-off values of the baseline TyG-BMI are ≤254.12, 254.12~291.61, 291.61~338.70 and ≥338.70. The quartile cut-off values of the baseline TyG-WC are ≤913.82, 913.82~1005.50, 1005.50~1121.59 and ≥1121.59. The quartile cut-off values of the baseline TyG-WHtR are ≤5.53, 5.53~6.07, 6.07~6.74 and ≥6.74. Crude model adjusts for: none. Model 1 adjusts for: age (years); gender; race (white; black; other) and education (<high school; ≥high school); PIR. Model 2 adjusts for: age (years); gender; race (white; black; other); education (<high school; ≥high school); PIR; HDL; LDL; TC; hypertension (yes or no) and retinopathy (yes or no). * *p* < 0.05, ** *p* < 0.01.

**Table 3:** Subgroup analysis of the associations between the TyG index and diabetic retinopathy.

Subgroup	OR (95%CI)	*p*	*p* for Interaction
Age (years)			0.055
≤62	2.193 (1.251–3.846)	0.006 **	
>62	1.738 (0.919–3.286)	0.089	
Gender			0.013 *
Male	1.380 (0.778–2.446)	0.27	
Female	2.669 (1.395–5.109)	0.003 **	
Race			0.194
White	1.771 (0.852–3.691)	0.125	
Black	1.881 (0.902–3.926)	0.092	
Other	2.530 (1.162–5.530)	0.019 *	
Education			0.179
≤high school	2.516 (1.222–5.178)	0.012 *	
>high school	1.921 (1.134–3.254)	0.015 *	
PIR			0.874
≤62	1.349 (0.831–2.337)	0.208	
>62	3.698 (1.729–7.910)	0.001 **	
LDL(mg/dL)			0.655
<100	1.939 (1.063–3.538)	0.031 *	
≥100	2.063 (1.137–3.744)	0.017 *	
HDL(mg/dL)			0.608
<50	2.154 (1.217–3.811)	0.008 **	
≥50	1.850 (0.983–3.482)	0.056	
TC (mg/dL)			0.013 *
≤178	1.003 (0.999–1.007)	0.140	
>178	1.004 (1.001–1.006)	0.006 **	
Renopathy			0.032 *
Yes	2.096 (1.328–3.307)	0.001 **	
No	2.098 (0.623–7.006)	0.233	
Hypertension			0.726
Yes	1.971 (1.174–3.310)	0.010 *	
No	1.839 (0.865–3.910)	0.114	

Abbreviations: PIR poverty income ratio, HDL high density lipoprotein, LDL low density lipoprotein, TC total cholesterol, * *p* < 0.05, ** *p* < 0.01.

**Table 4:** Vasculopathy subgroup analysis of the associations between the TyG index and diabetic retinopathy.

Parameter	Vasculopathy OR(95%CI), *p* Value	Non-Vasculopathy OR(95%CI), *p* Value
TyG index	0.520 (0.197–1.372), 0.187	2.656 (1.643–4.294), <0.001 **
TyG-WC	1.001 (0.998–1.003), 0.547	1.000 (0.999–1.001), 0.714
TyG-BMI	1.003 (0.998–1.009), 0.227	0.999 (0.997–1.002), 0.645
TyG-WHtR	1.128 (0.753–1.691), 0.559	1.017 (0.833–1.241), 0.868

Abbreviations: TyG triglyceride–glucose, BMI body mass index, WC waist circumference, WHtR waist-to-height Ratio, ** *p* < 0.01.

## Data Availability

The data used in the present research were obtained from publicly accessible sources. These data could be accessible at the following URL: https://www.cdc.gov/nchs/nhanes/ (accessed on 29 October 2022).
